# Effect of 5.25% Sodium Hypochlorite Enamel Deproteinization on the Initial Shear Bond Strength and Resin Penetration in the Enamel of Orthodontic Brackets Bonded With a Self‐Etching Primer: An In Vitro Study on Human Bicuspids

**DOI:** 10.1155/ijod/8861250

**Published:** 2026-07-29

**Authors:** Nadia Kebaïli, Manon Fourneron, Myriam Mati, Hamideh Salehi, Stéphane Barthelemi, Ziad Tannouri

**Affiliations:** ^1^ Université de Reims Champagne Ardenne Bibliothèque Universitaire, Reims, France; ^2^ Université de Montpellier Faculté d’Odontologie, Montpellier, France; ^3^ ICube, UMR 7357, CNRS, INSERM, University of Strasbourg, Strasbourg 67000, France, unistra.fr

**Keywords:** adhesive remnant index, deproteinization, orthodontic brackets, Raman confocal microscopy, shear bond strength

## Abstract

**Objective:**

This study aimed to evaluate the effects of enamel deproteinization (DP) on the shear bond strength (SBS) and on bracket/adhesive failure mode of brackets bonded with a self‐etching primer (SEP). A secondary objective was to assess resin penetration into enamel using Raman microscopy across the three bonding protocols.

**Materials and Methods:**

Seventy‐eight extracted human bicuspids were divided into three groups (*n* = 26): Group A, brackets were bonded on enamel pretreated with 5.25% sodium hypochlorite (NaOCl) and SEP, Group B with SEP without DP, and Group C (control), with a conventional 37% phosphoric acid etching protocol. SBS was measured, and the debonding characteristics were assessed with the adhesive remnant index (ARI). SBS was analyzed using the Kruskal–Wallis test with post hoc Wilcoxon tests and Bonferroni correction, while ARI was analyzed using Fisher’s exact test. For resin penetration evaluation, an additional 12 bicuspids were prepared using the same protocol and analyzed by Raman confocal microscopy. Data were analyzed using a mixed model for repeated measures.

**Results:**

DP with 5.25% NaOCl increases SBS of the SEP, reaching values close to the conventional bonding protocol (H_3_PO_4_: 121.16 MPa; DP + SEP: 119.92 MPa; SEP alone: 80.24 MPa; *p*  < 0.001). ARI scores comparison showed more residual adhesive remaining on teeth pretreated with NaOCl (ARI 3%:54%) compared to SEP alone (ARI 3%:12%) and Reference (ARI 3%:81%; *p*  < 0.001). Raman confocal microscopy revealed the deepest resin penetration in the control group, whereas the NaOCl + SEP and SEP‐alone groups exhibited comparable penetration depths.

**Conclusion:**

This study demonstrated that enamel DP enhances bracket bond strength with SEP, reaching a resistance close to conventional bonding with orthophosphoric acid etching. Despite similar strength, deproteinized enamel exhibited less resin penetration than conventionally etched enamel.

## 1. Introduction

Phosphoric acid etching is the most common method used for enamel surface preparation prior to orthodontic bracket bonding. But for many years now, a new generation of adhesives has been available: self‐etching primers (SEPs), which mix etching and priming agents into a single acidic primer solution that etches and primes simultaneously. Combining them into a single treatment step is likely to reduce clinical chair time and improve cost‐effectiveness [1, 2].

Although many studies have shown that SEPs provide the same shear bond strength (SBS) as the conventional 3‐step adhesive technique [3–6], some researchers suggest, if additional SBS is desired, preconditioning the enamel surface by burring, acid etching, or sandblasting [7, 8].

Espinosa et al. in 2008 [9] suggested that the use of 5.25% sodium hypochlorite (NaOCl) for 60 s as a deproteinizing agent before acid etching would increase the bond strength. Indeed, organic elements would be better removed, both from the enamel structure and from the acquired film, and the enamel‐retentive surface and Silverstone’s type I and II etching patterns are increased.

In another study, Espinosa et al. [10] concluded that enamel pretreatment with 5.25% NaOCl for 60 s before acid etching significantly increases the quality and depth of the resin replica which could significantly increases the retention of all adhesive restorations.

Justus et al. in 2010 [11] confirmed clinically those results, indicating that deproteinization (DP) with 5.25% NaOCl for 60 s significantly increases the SBS of brackets bonded with Fuji Ortho LC resin‐modified glass ionomer (RMGI). In 2012 and 2013, Pithon et al. [12, 13] studied the effect of 8% and 10% papain gel enamel DP before bracket bonding with RMGI and concluded that DP increases the SBS. More recent studies confirmed DP efficiency for bracket bonding resistance [14, 15] and even with fluorosed enamel [16].

Noninvasive, label‐free confocal Raman microscopy measures the inelastic scattering of incident light energy to analyze the structure of mineralized tissues as well as resin. The spatial resolution of Raman microscopy (300 nm) provides a high‐resolution chemical map of phosphate, carbonate, and organic structure, and all resin chemical signatures. Confocal Raman microscopy helps to trace the penetration of resin/glue in enamel after different surface treatments with no need to dye or marker to predict greater bracket bonding resistance.

Previous studies using Raman microscopy have investigated the effects of different bonding protocols on eroded dentin (Sirirangsee et al. [17], fluorotic enamel (Huilcapi et al. [18]), and hypomineralized enamel (Natarajan et al. [19]). However, to the best of our knowledge, no research has yet evaluated resin penetration after DP in physiological enamel.

The primary objective of this study was to evaluate the effect of 5.25% NaOCl enamel DP on the initial SBS of orthodontic brackets bonded with a SEP. The primary null hypothesis was that enamel DP does not result in a statistically significant difference in SBS compared with SEP alone or conventional phosphoric acid etching. The secondary objective was to assess resin penetration into the enamel using Raman confocal microscopy. The secondary null hypothesis was that there was no statistically significant difference in resin penetration depth among the three bonding protocols.

## 2. Material and Methods

### 2.1. Sample Preparation

#### 2.1.1. Bonding Preparation

Seventy‐eight freshly extracted human (48 first or second maxillary and 30 first or second mandibular) bicuspids extracted for orthodontic reasons were collected after patient consent, cleaned of calculus and soft tissues, and stored in saline solution at room temperature for a maximum period of 6 months with biweekly saline replacement. All patients gave their written consent for teeth extraction and their use for experimentation (IRB Agreement Number: IRB‐MTP‐2021‐100‐927).

The criteria for tooth selection were intact buccal and lingual enamel, no use of previous preconditioners or chemical agents such as hydrogen peroxide (H_2_O_2_), no cracks caused by extraction forceps, and absence of caries, hypoplasia, white spots, abrasion, or colorations.

The premolars were randomly divided into three groups (*n* = 26) based on the enamel pretreatment protocol before bonding: 5.25% NaOCl DP followed by Transbond Plus SEP (3M Unitek, Monrovia, CA, USA) (Group A), Transbond Plus SEP (Group B), and 37% phosphoric acid etching followed by bonding with Transbond XT primer and resin (Group C).

Each group included 26 teeth with an equal number of first, second, maxillary, and mandibular premolars in each group to prevent bias caused by possible differences in bond strength among tooth types.

The buccal surfaces of all teeth were cleansed with nonfluoridated, oil‐free pumice paste (Prophylaxe Paste, Détartrine 150 Z, Septodont, Saint Maur, France) applied with a prophylactic brush on a slow‐speed handpiece for 10 s, rinsed with water for 10 s, and dried with an oil‐and moisture‐free air spray for 10 s. Then, the brackets were treated according to the bonding protocol of each group:

Group A (DP + SEP): The enamel surface was treated with 5.25% NaOCl applied with a sterile cotton pellet for 60 s, rinsed for 10 s, and dried with an oil‐ and moisture‐free air spray for 10 s. Then, the SEP was rubbed on the enamel surface for 5 s according to Büyükyilmaz et al. [5]. Orthodontic metal brackets (maxillary right first premolars, serial N° 350–0370, Mini Diamond Twin, Ormco Corporation, Glendora, Calif., USA) were bonded on all the samples with the same composite resin (Transbond XT). Each bracket was placed on the enamel with a compressive force, and any excess resin was removed with a small scaler.
Group B (SEP): The SEP protocol was identical as applied to group A but without DP.
Group C (H_3_PO_4_): The enamel was etched with 37% phosphoric acid (Total Etch, Ivoclar‐Vivadent, Schaan, Liechtenstein) applied for 30 s and then rinsed with water for 30 s. The enamel was then dried with an oil‐and moisture‐free air spray and inspected to ensure a chalky, frosty appearance, and a thin layer of Transbond XT primer was applied with a microbrush for 5 s, and the brackets were bonded in the same conditions as in Groups A and B.

Each tooth was then light‐cured for 10 s mesially and 10 s distally with a 3000 mW/cm^2^ LED curing light (Mini LED Ortho 2, Satelec, Merignac, France). Once the resin was cured, the roots of all the teeth were embedded in individual 40 mm x 40 mm cold‐cure acrylic resin (ProBase Cold, Ivoclar Vivadent, AG, Schaan, Liechtenstein) bases.

**Figure 1:**
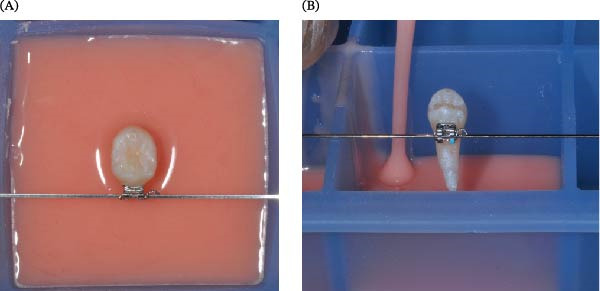
Bracket positioning in the resin block with a SS .018x.025 wire for standardization, (A) occlusal view, (B) buccal view.

The enamel‐bracket interface was monitored for alignment with the force vector of the universal testing machine. To ensure the interface was as vertical as possible, each tooth was carefully positioned in its mold by ligaturing a 0.018 × 0.025 SS (stainless steel) wire in the bracket and placing this wire on the edge of the square silicon mold (Figure 1). After the acrylic resin had cured, all the samples were stored in deionized water at room temperature. The SBS experimentation was performed 24 h after bonding according to Turk et al. [20] recommendations.

#### 2.1.2. SBS Analysis

**Figure 2:**
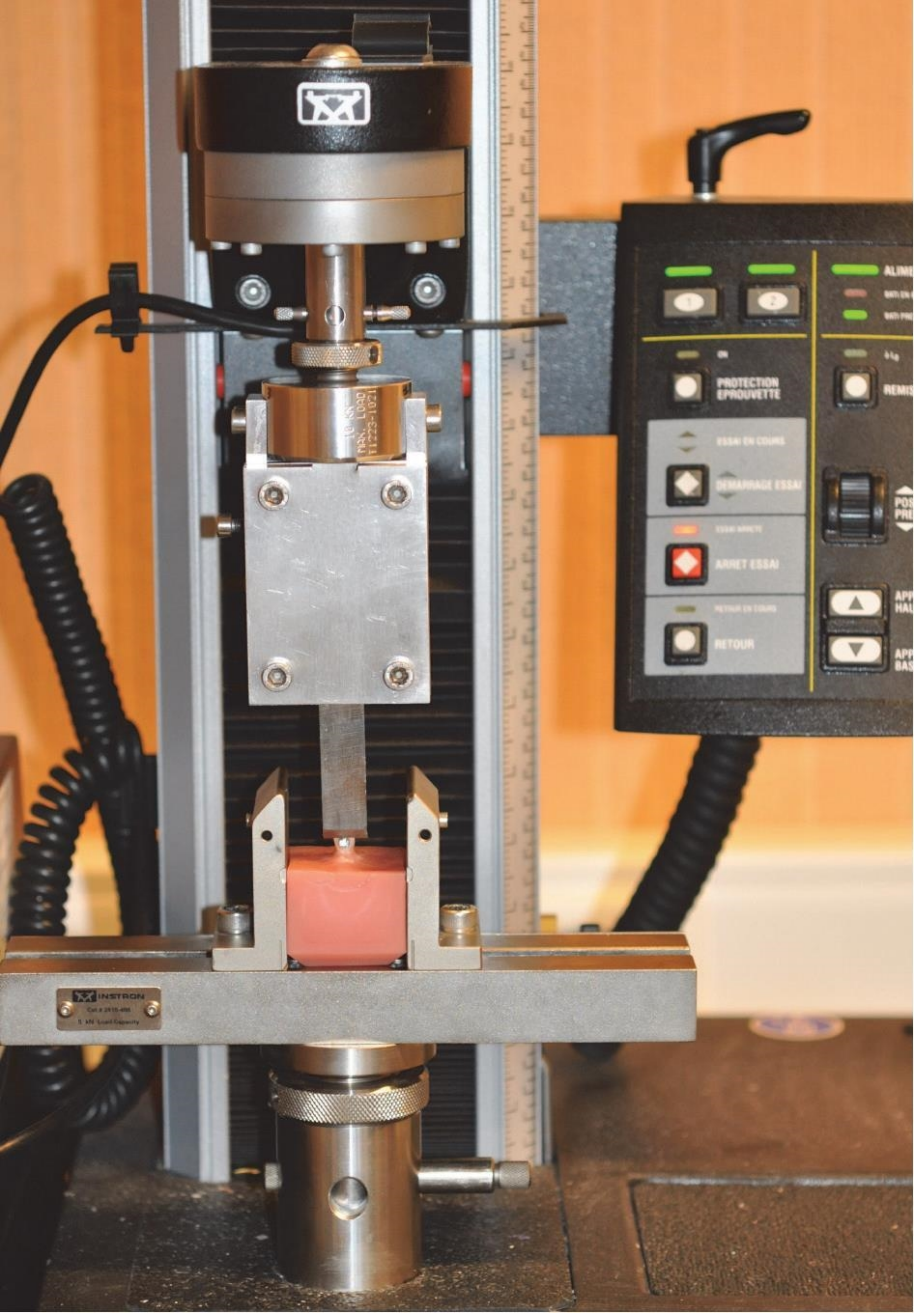
Instron machine model 5544 for SBS experimentation.

SBS was measured using a universal testing machine (Model 5544; Instron Ltd., High Wycombe, Buckinghamshire, UK) at a crosshead speed of 1 mm/min. Each tooth along with its resin base was fixed in the clamping fixture so that the surface to be tested was parallel to the force during the shear strength test. An occluso‐gingival loading was applied to the bracket, producing a shear force at the bracket‐tooth interface (Figure 2). The Bluehill 2, Version 2.5 (Instron) software connected to the testing machine recorded the results from each test in Newtons (N).

The enamel surface was examined under 10x magnification by two independent observers to determine how much residual adhesive remained on the tooth. The debonding characteristics of each specimen were established using the adhesive remnant index (ARI) developed by Artun and Bergland [20]. ARI scores range from 0 to 3: 0 = no residual adhesive on the enamel; 1 = less than half of the adhesive remaining on the tooth surface; 2 = more than half of the adhesive remaining on the tooth; and 3 = all adhesive remaining on the tooth, with a distinct impression of the bracket base.

### 2.2. Statistical Analysis

The initial objective was to identify a large difference (Cohen’s *d* = 0.8) between the SEP and SEP + DP groups with 80% power and an alpha risk of 5%. The calculation yielded 26 individuals per group.

Since the variables SBS and ARI do not follow a normal distribution, according to the Shapiro test, nonparametric tests were performed: the Kuskal–Wallis test for comparing mean ranks for SBS and the Wilcoxon signed‐rank test with Bonferroni correction for pairwise comparisons. Fisher’s exact test was also performed to compare the distribution of ARI according to the treatment, considering ARI as a categorical variable.

A linear mixed model was used to compare the average resin penetration in the three groups, considering repeated measurements.

All tests and tables were performed using R software Version 4.5.1 with the gtsummary package.

### 2.3. Raman Confocal Microscopy Sample Preparation

To evaluate resin penetration into the enamel surface using the three different bonding protocols, 12 freshly extracted human premolars were collected from three different patients (4 premolars per patient: teeth 15, 25, 35, and 45). The premolars from each patient were assigned to one of three experimental groups: Group A (DP and SEP protocol), Group B (SEP protocol), and Group C (37% phosphoric acid etching followed by bonding with Transbond XT primer). All specimens were conditioned and prepared under the same conditions as those used for the SBS experiment.

**Figure 3:**
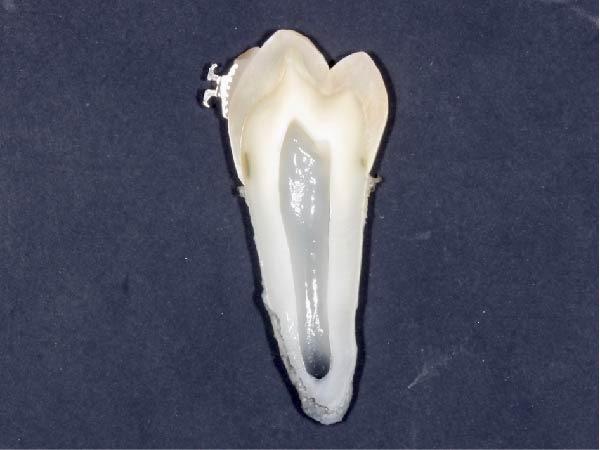
Sample preparation for confocal Raman microscopy.

All samples were stored in saline solution at room temperature before Raman confocal microscopy preparation. Samples were sliced in the sagittal plane (Saw Isomet 2000, Buehler, Lake Bluff, USA), and one of the surfaces of each slice was polished and cleaned by ultrasonic baths to obtain a very smooth plane to get a good Raman signal (Figure 3); then, the samples were analyzed with Raman confocal microscopy. To collect the Raman spectra, a Witec Confocal Raman Microscopy α300R (Witec, Ulm, Germany) was used. Data acquisition was performed using Image Plus 2.08 software from Witec. Data analyses are based on two methods, K‐means cluster analysis (KMCA) and the Pearson correlation coefficient. The first method, KMCA, partitions data into K mutually exclusive clusters. The K‐mean treats each spectrum as an object located in a multidimensional space. The objects in each cluster are as close to each other as possible in each partition. KMCA was realized using Witec Project Plus (Ulm, Germany) software. Calculation of the spectral correlation matrix as the second method confirms the results of KMCA and helps the calculation and interpretation of resin penetration. The most similar spectrum to the reference spectrum of resin has been calculated to quantify the similarity, as a “distance,” using the Pearson’s correlation coefficient. The results are expressed as a percentage from −100% (perfect negative correlation) to 100% (perfect positive correlation), and a pseudo‐color map is produced. Pearson correlation calculations were performed with a homemade code written in MATLAB (MathWorks, Inc., Natick, MA, USA).

**Figure 4:**
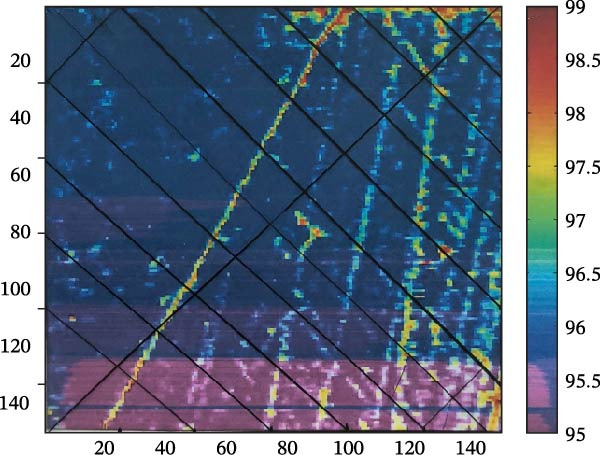
Confocal Raman microscopy image with grid for resin penetration evaluation.

To assess the mean thickness of bonding penetration into the enamel, a calibrated grid including 11 equal zones was created and positioned on the area to be analyzed (Figure 4). To minimize the measurement error method, the thickness was assessed n a randomized zone through the 11 zones determined by the grid, and the measurements were triplicated.

## 3. Results

SBS assessment using the Kruskal–Wallis test showed statistically significant differences among the three groups (*p*  < 0.001). Post hoc pairwise comparisons using Wilcoxon tests with Bonferroni correction revealed no statistically significant difference between Group C (H_3_PO_4_) and Group A (DP + SEP), whereas both Group C and Group A demonstrated significantly greater SBS values than Group B (SEP) (*p*  < 0.001). Table 1 presents the mean values (SD) for the SBS of the three groups.

**Table 1:** Descriptive statistics and comparison of shear bond strength (SBS) among the three groups.

Characteristic	Reference (H_3_PO_4_) *N* = 26^1^	SEP *N* = 26^1^	DP + SEP *N* = 26^1^	*p*‐Value^2^
SBS	121.16 (48.63)^a^	80.24 (24.17)^b^	119.92 (32.22)^a^	<0.001

*Note*: Post hoc Wilcoxon tests with Bonferroni correction: groups sharing a letter in a row are not significantly different.

^1^Mean (SD).

^2^Kruskal–Wallis rank sum test.

**Figure 5:**
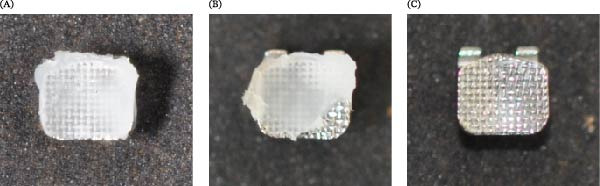
ARI scores: (A) score 0, (B) score 1, (C) score 2.

Fisher’s exact test showed significant differences in ARI score distribution among the three groups (*p*  < 0.001). Group B (SEP) specimens predominantly exhibited an ARI score of 0, indicating adhesive bond failure at the enamel/composite interface, whereas Groups C (H_3_PO_4_) and A (DP + SEP) mainly showed an ARI score of 3, indicating cohesive bond failure with composite resin remaining on the enamel (Figure 5). The ARI scores are listed in Table 2.

**Table 2:** Distribution of adhesive remnant index (ARI) scores according to treatment group.

	ARI				
	0	1	2	3	Total
Group					
Reference	0 (0%)	0 (0%)	5 (19%)	21 (81%)	26 (100%)
SEP	16 (62%)	7 (27%)	0 (0%)	3 (12%)	26 (100%)
DP + SEP	2 (7.7%)	5 (19%)	5 (19%)	14 (54%)	26 (100%)
Total	18 (23%)	12 (15%)	10 (13%)	38 (49%)	78 (100%)

*Note:* Fisher’s exact test, *p*  < 0.001. 0 = no residual adhesive on the enamel; 1 = less than half of the adhesive remaining on the tooth surface; 2 = more than half of the adhesive remaining on the tooth; 3 = all the adhesive remaining on the tooth, with a distinct impression of the bracket base.

**Figure 6:**
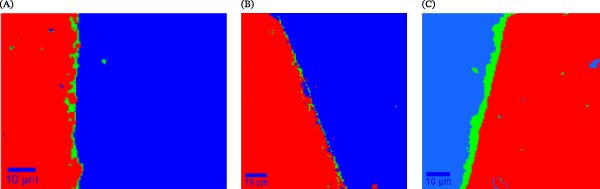
Confocal Raman microscopy images obtained from KMCA method: blue = enamel, red = resin, Green = resin penetration; (A) = DP+SEP sample, (B) = SEP sample, (C) = 37% H3PO4 etching and bonding protocol.

The confocal Raman microscopy revealed statistically significant differences between the groups using repeated measures analysis concerning resin penetration: in Group A (DP + SEP), the mean enamel penetration was 2.1 μm (Figure 6A), while in Group B (SEP), the mean value was only 0.8 μm (Figure 6B). For Group C (H_3_PO_4_), the mean value was 12.4 μm (Figure 6C). The linear mixed model is presented in Table 3.

**Table 3:** Linear regression analysis of resin penetration thickness (µm) according to treatment group.

Characteristic	Resin penetration (µm)	Beta	95% CI	*p*‐Value
Group				
Reference	12.4	—	—	—
SEP	0.8	−11.6	−15.0, −8.30	<0.001
DP + SEP	2.1	−10.3	−13.7, −6.96	<0.001

Abbreviation: CI, confidence interval.

## 4. Discussion

This study compared the initial SBS with three different bonding protocols to assess the efficacy of 5.25% NaOCl DP to increase the bracket resistance to debonding with a SEP (Group A) in comparison with the SEP protocol without DP (Group B) and the classic bonding protocol with 37% H_3_PO_4_, considered the gold standard (Group C). SBS differed significantly between Groups A and B (*p*  < 0.001), while no significant difference was found between Groups A and C based on post hoc Wilcoxon comparisons with Bonferroni adjustment.

The results demonstrated that the classical etching protocol with 37% H_3_PO_4_ (Group C) gives a significantly better resistance to SBS in comparison to the SEP protocol (Group B) in accordance with the experimentation of Aljubouri et al. [21] and Yamada et al. [22]. The results found that SBS was significantly increased when 5.25% NaOCl DP was performed before SEP (Group A) in comparison to Group B, confirming that the 1‐min DP protocol significantly increases the SBS. Our results corroborate those of Espinosa et al. in 2008 [9] and 2010 [10] as they demonstrated that DP increases the Silverstone type I and II surface attack on the enamel and increases the depth of enamel pits, allowing a stronger bonding. Indeed, DP allows the acid monomers of the SEP to penetrate more easily and deeper into the enamel surface. Mechanical retention is enhanced. Another study performed by Hamdane et al. [23] demonstrated an increased adhesion of bonded brackets with glass ionomer cement (GIC) when a DP with a 5.25% NaOCl solution was applied under the same conditions as in the present study before the bonding protocol. More recently, Mahmoud et al. [14] and Panchal et al. in 2019 [15] also demonstrated the benefit of 5.25% NaOCl DP in bracket adhesion. Conversely, Daou et al. in 2021 [24] found that enamel DP with 5.25% NaOCl, regardless of application time, did not significantly affect the SBS of the SEP Transbond Plus. Pereira et al. [25] found that enamel DP with 5.25% NaOCl increased bracket bond strength when using GIC and RMGI cement (RMGIC), but the improvement was not statistically significant compared to untreated enamel.

The current study found no statistically significant difference in SBS between Group A and Group C, indicating that DP increases bracket adhesion when using the SEP protocol, reaching adhesive values comparable to those obtained by the conventional 37% H_3_PO_4_ etching and bonding protocol.

Moreover, the results found a statistically significant difference in the ARI score between the three tested groups. The high ARI scores in Groups A and C indicate that most adhesive remained on the tooth, which may suggest a lower risk of enamel damage during debonding. Conversely, the low ARI scores in Group B suggest that most adhesive remained on the bracket, potentially compromising the bonding efficiency.

The ARI scores in the different groups confirmed that DP allows an increase in primer adherence to the tooth. These results are in contradiction with the studies of Sharma et al. [26] and Mahmoud et al. [14], who did not find any statistically significant difference in ARI scores with or without DP before bonding. Alternatively, Ghoubril et al. [27] found that the V‐Prep: hypochlorite/orthophosphoric acid mixture enhanced RMGIC bonding, making it suitable for palatal or lingual and hard‐to‐dry surfaces. However, further research is needed before recommending RMGIC bonding over resin composite for buccal surfaces.

Resin penetration is a key factor for bracket adhesion and resistance. On the other hand, enamel demineralization should be the lowest possible to avoid permanent lesions on the enamel. Moreover, when brackets are bonded, the total etched enamel area generally exceeds the bracket base area, leading to a portion of enamel not being covered by the bracket but only by the bonding primer. The balance between the etching depth and bracket resistance must be considered. In the present study, the Raman confocal microscopy revealed statistically significant differences for resin penetration within the three groups. Predictably, the conventional 37% H_3_PO_4_ etching and bonding group (Group C) showed the deepest resin penetration in the enamel (12.4 μm), associated with the highest SBS score, indicating greater bracket bond resistance. The SEP group (Group B) revealed the lowest resin penetration in the enamel (0.8 μm) associated with the lowest SBS score. Finally, the DP + SEP Group (Group A) revealed a slightly greater resin penetration in the enamel than the Group B (2.1 μm) associated with an SBS value comparable to that of Group C. Although conventional phosphoric acid etching produced substantially deeper resin penetration, both the DP + SEP and the conventional etching groups achieved SBS values within clinically acceptable ranges. This finding suggests that increased penetration depth does not necessarily translate into superior clinical performance. While deeper resin infiltration may enhance micromechanical retention, excessive enamel demineralization could potentially increase the risk of enamel alteration during debonding. Therefore, achieving adequate bond strength with more conservative penetration depths may represent a clinically relevant balance between effective bonding and the preservation of enamel integrity.

A limitation of this study is the small sample size for the Raman microscopy component, with only 12 teeth analyzed (four per group). Serving as a pilot, it represents the first reported use of Raman confocal microscopy to compare multiple bonding protocols in the literature, and the labor‐intensive specimen preparation limited the sample size. Nevertheless, these results provide preliminary data and a methodological framework for future large‐scale investigations.

Using scanning electron microscopy (SEM) observation, Mahmoud et al. [14] found differences in depth of resin penetration with a deeper penetration in the enamel when DP was performed before bonding whatever the type of resin used. These results confirmed the advantage of 5.25% NaOCl DP as it increases bracket resistance to breakage without inducing a major increase in resin penetration in the enamel as the latter is only doubled between SEP and DP + SEP, while this rate is multiplied by five between the DP + SEP group and the 37% H_3_PO_4_ etching and bonding group.

Using Raman microscopy, Sirirangsee et al. [17] demonstrated that pretreatment with the papain enzyme can enhance and sustain the bonding performance of self‐etch adhesives on eroded dentin. Huilcapi et al. [18] reported that applying NaOCl or sandblasting in combination with phosphoric acid enhanced the SBS of brackets on fluorotic enamel, without affecting the cohesive durability of the resin cement, and improved enamel interprismatic conditioning. Moreover, Natarajan et al. [19] used Raman spectroscopy to assess the effects of different pretreatments (hydrochloric acid [HCl], NaOCl, H_2_O_2_) on resin infiltration in molar–incisor hypomineralization (MIH). They found that these protocols increased the infiltrant penetration depth and made the treated MIH enamel structure more similar to normal enamel, indicating reduced protein content after treatment.

## 5. Conclusion

This in vitro study showed that enamel surface DP with 5.25% NaOCl for 60 s prior to bracket bonding was associated with an increase in SBS when used in combination with a SEP, along with a reduction in resin penetration into the enamel.

Raman confocal microscopy analysis demonstrated that conventional 37% phosphoric acid etching produced the greatest resin penetration, while the SEP, whether applied alone or following DP, resulted in reduced penetration depths.

## 6. Recommendations

Further in vitro investigations incorporating artificial aging methods, such as thermocycling or long‐term water storage, are required to better assess the durability of the DP protocol over time. Additionally, future studies should evaluate the influence of different NaOCl concentrations and application times on the enamel‐bonding performance.

Well‐designed in vivo studies, including controlled clinical trials such as split‐mouth designs, are necessary to test the clinical efficiency of the DP protocol for bonding.

## Funding

No funding was received for this manuscript.

## Conflicts of Interest

The authors declare no conflicts of interest.

## Data Availability

The data that support the findings of this study are available from the corresponding author upon reasonable request.
